# Soybean hull inclusion on gilt growth and first parity total born

**DOI:** 10.1093/tas/txag169

**Published:** 2026-10-22

**Authors:** Mariah L Mayer, Robert J Harrell, Laura L Greiner

**Affiliations:** Department of Animal Science, Iowa State University, Ames, IA 50011, United States; Passel Farms, Osceola, IA 50213, United States; Department of Animal Science, Iowa State University, Ames, IA 50011, United States

**Keywords:** gilt development, soy hulls, swine

## Abstract

Four thousand three hundred and forty-six gilts from 10 gilt developer rooms (DNA Line 241, DNA Genetics, Columbus, NE) were weaned at ∼21 days and assigned to either a control (CON) diet based on corn and soybean meal or a slow growth (SG) diet containing increasing levels of soybean hulls (up to 50% soybean hulls) starting at approximately 25 kg of body weight to evaluate if feeding increasing levels of soybean hulls (dietary fiber) would reduce gilt growth rate and improve first parity performance. Each room was an experimental unit. Feed and water were provided ad libitum. Growth, puberty indicators, and reproductive traits were assessed through first parity. Subsets of gilts were vulva scored (day 111 of age; 150 gilts per treatment), blood was sampled for hormone analysis (days 150 and 164 of age; 40 gilts per treatment), and evaluated by ultrasound for back fat (BF) and loin depth (LD). Data were analyzed in SAS 9.4 using the GLIMMIX procedure, with cohorts as the experimental unit. Gilts fed the CON diet were heavier (102.2 vs. 94.8 ± 2.26 kg; *P* < 0.01), had wider vulvas, and greater back fat (15.1 vs. 12.5 mm ± 0.28; *P* < 0.01) compared to SG gilts. Loin depth did not differ between treatments. Age at first breeding was slightly younger for CON (227.7 vs. 231.8 ± 4.09 days; *P* = 0.44), though not significant. Total pigs born (15.0 and 14.8 ± 0.21 pigs/sow, respectively) and weaned (13.0 and 12.9 ± 0.24 pigs per sow, respectively) at first parity were unaffected by diet. Feeding soybean hulls to growing gilts reduced growth rates and back fat delaying puberty onset without impacting first-parity total born and pigs weaned performance. These findings suggest soybean hulls can be a tool for managing gilt growth rates without compromising productivity.

## Teaser

Feeding increasing concentrations of soybean hulls to growing gilts reduced growth rates and back fat delaying puberty onset without impacting first-parity total born and pigs weaned performance.

## Introduction

Current trends in the swine industry indicate a rise in sow replacement rates, which is associated with poor longevity and results in substantial economic implications including increased production costs (Eckberg 2021). Sows and gilts are culled or removed from herds due to health issues, poor performance, or management decisions (Calderón Díaz et al. 2015). The average culling rate is ∼ 50% with mortality rates ∼ 14% (Hoge and Bates 2011; Mote et al. 2009; Eckberg 2021). Reasons for culling vary and include reproductive failure, low litter size, peripartum disorders, low milk production, and locomotion disorders (Anil et al. 2005; Zimmerman 2012; Mote et al. 2015). Removal of parity 0 and 1 females results in a lower herd parity average and a reduced lifetime number of pigs weaned per sow per year compared to those herds that retain more of the parity 2, 3, or 4 sows, reducing the probability of reaching peak production and causing financial loss (Stalder et al. 2003; Anil et al. 2005).

A primary objective of gilt nutrition for enhancing their longevity is to develop prepubertal gilts during a period of rapid physiological growth of body weight (BW), tissue composition, and bone and reproductive development. Maintaining growth rates to achieve 115 to 140 kg at puberty (150 to 220 days of age) and 135 to 160 kg at breeding is crucial to avoid locomotion and structural issues leading to early removal from the herd (Soede et al. 2011; Marchant 2015; Faccin et al. 2022). However, older research would suggest that restricting energy alters lean growth and increased bone length, cortical thickness and percentage of ash increased due to an increase in age versus a set weight (Robbins et al. 2002).

Overall, higher inclusion of fiber in the diet will decrease energy concentration per kg of diet (Wenk 2001). Feeding a higher fiber diet also results in an earlier satiety due to stomach wall elongation and gastrointestinal expansion (Kerr and Shurson 2013). Previous research demonstrated that gilts housed in group pens fed a diet high in fiber during development had lower feed intake and thus lower body weight and back fat compared to those on control diets indicating that feeding fiber in gilt development could be beneficial in slowing the growth rate of gilts (Gregory et al. 2023). The collective work on feeding high fiber in the gilt developer phase has demonstrated the value of fiber in slowing gilt growth rates; however, data on the impact of fiber delaying puberty and altering ovulation rates is mixed (Klindt et al. 2001; Calderón Díaz et al. 2015; Johnson et al. 2022). Alternating a low-energy diet with fiber and a high-energy diet versus a control group continuously fed a high-energy slowed gilt growth rate, but reduced pre-lactation mammary development (Lyvers-Peffer and Rozeboom 2001).

Little work has provided the impact of fiber on gilt retention and reproductive performance such as total born and embryo survival (O’Bannon et al. 1966; Johnson et al. 2022). While insoluble dietary fiber in the form of dried distiller’s grain is readily available and commonly used in the United States, the data supports the benefits of soluble fiber sources. A common source of soluble fiber that is available and potentially economical for use in swine diets is soybean hulls. Therefore, the objective of this study was to determine if increasing soybean hull concentration and fiber concentration in the diet would reduce pre-pubertal growth rate, improve first-parity retention and total born. The hypothesis of this study was that the inclusion of soybean hulls in gilt development diets would slow growth, delay pubertal onset, and increase gilt total born and longevity through parity 1.

## Materials and methods

All experimental protocols adhered to guidelines for the ethical and humane use of animals for research according to the Guide for the Care and Use of Agricultural Animals in Research and Teaching (FASS 2010) and were approved by the Institutional Animal Care and Use Committee at Iowa State University (IACUC 22–210).

### Animal, housing and experimental design

This experiment was conducted at a commercial gilt development unit (Osceola, IA) starting February 2023 and ending December 2023. The facility contained a series of nursery rooms and grow-finish rooms. Each month, approximately 1000 weaned gilts (approximately 21 days of age; DNA Line 241, DNA Genetics, Columbus, NE) entered one of the 1000 head nursery rooms and the gilts would remain in the rooms for approximately 8 weeks. At the end of the nursery period, gilts were moved to one of five 1000 head grow-finish rooms connected within the gilt developer unit. The gilts would remain in the assigned room until the gilts were sent to a sow farm as a sexually mature replacement female. Each room was managed similarly to an individual grow-finish barn. Additionally, each grow-finish room had two tandem feed bins that fed only that room resulting in one dietary treatment being assigned to each room.

Within each room, gilts were housed in small pens (30 to 35 gilts). Each nursery pen contained a dry feeder and two water nipples to allow ad libitum intake of both feed and water. The grow-finish rooms housed similar numbers of gilts and each pen was equipped with one wet-dry feeder (two feeder holes), and one water nipple which allowed ad libitum feed and water intake. Boar exposure and heat check started at 23 weeks of age and continued weekly until gilts were moved out of the facility.

For the purpose of the study, due to the arrangement of the feeding system, one grow-finish room could only receive one dietary treatment; thus, at the time of arrival into the grow-finish period, each group of gilts was assigned a single treatment. Treatments were assigned as every other room. The trial started in February of 2023 with the first grow-finish room being assigned as the slow growth program. The following month, the next grow-finish room filled was assigned to the control program. These two rooms created the first cohort (replication of the study). This assignment of groups continued through the trial period. In order to allow for adequate replication, 10 rooms of gilts were used for the study resulting in approximately 10,000 gilts starting the dietary treatments (5 replications per treatment). A cohort was consider a single replication of the study (one room of the control and one room of the dietary treatment). At the completion of the gilt developer period, gilts within each cohort were randomly assigned across multiple sow farms and each treatment was represented within each sow farm over time. Gilt identification numbers were tracked from the gilt developer unit to the sow farms, so that reproductive performance metrics could be tracked. Animals were removed from study early due to downstream gilt needs due to disease outbreaks or mortality, and were removed at the time of selection due to culling associated with poor anatomical structure for replacement gilts resulting in 4346 gilts completing the study. On average, each room had approximately 6% culls, 4% mortality, and marketed 3% of the remaining animals not selected for the farms. The animals removed early were selected by the farm staff and were selected across the room through the removal of whole pens in order not to influence space allocation and to optimize the efficiency of the feeders. The number of animals removed were recorded for each room. One room per treatment group was heavily impacted (>1000 animals per room) and were removed from the study data, and two cohorts (two rooms per treatment) were moderately impacted (∼ 600 animals per room) by the early movement of animals to sow farms to address herd closures. Animals were not re-arranged in the rooms in the middle of the grower period to minimize injury and aggression.

### Dietary treatments

Pigs were fed a common diet in the nursery which consisted of four dietary phases without soy hulls each lasting approximately 10–12 days. Once moved to the grower-finisher, each cohort was assigned to one of two dietary treatments (control (CON) or slow growth (SG)). Dietary assignments to gilt developer unit (GDU) rooms were alternated between the SG and CON per group to minimize seasonal effects. For example, the first group was provided the SG assignment, the next month the new group received the CON and this created one cohort. The first room assignment for the trial was done by computerized randomization.

Within each phase, the two dietary treatments (CON or SG) were fed for approximately 4 weeks each and cohorts remained on the same treatment assignment for the remainder of the trial. The CON diet consisted of corn and soybean meal and the SG diet consisted of increasing soybean hull levels (12.5, 25, 37.50 and 50% inclusion of soybean hull reducing total dietary net energy (NE; Table 1). The formulated standard ileal digestibility (SID) Lys:NE was identical within dietary treatments across production phases. The SG diets were formulated to have a lower lysine density than the control diets; however, the diets were balanced on the SID Lys:NE. The standardized ileal digestible (SID) Lys value of the SG diets was formulated to meet the nutrient requirements of swine and the CON diet levels of SID Lys were above the requirement (National Research Council (NRC) 2012). All other nutrients met or exceeded the nutrient requirements identified in the NRC (2012).

**Table 1:** Ingredient composition for each dietary treatments (CON or SG) for gilts fed low levels of soybean hulls (CON (0%) or high levels of soybean hulls (SG (up to 50%)) in the gilt developer unit (GDU).

	Dietary phase							
	GDU1		GDU2		GDU3		GDU4	
Ingredients, %	CON	SG	CON	SG	CON	SG	CON	SG
**Corn**	63.40	55.60	68.10	49.50	73.50	44.50	77.40	34.40
**Soybean meal**	24.90	20.40	15.40	9.40	10.00	2.10	6.10	0.00
**Dried distiller’s grains with solubles**	7.50	7.50	12.50	12.50	12.50	12.50	12.50	12.50
**Soybean hulls**	0.00	12.50	0.00	25.00	0.00	37.50	0.00	50.00
**Soybean oil**	0.50	0.50	0.50	0.50	0.50	0.50	0.50	0.50
**Limestone**	1.10	0.90	1.10	0.60	1.00	0.40	1.00	0.30
**Monocalcium phosphate (21%)**	0.60	0.60	0.40	0.50	0.40	0.60	0.50	0.70
**Salt**	0.50	0.50	0.50	0.50	0.50	0.50	0.50	0.50
**Lysine—55%**	0.50	0.60	0.60	0.60	0.60	0.50	0.60	0.30
**Threonine^a^**	0.20	0.20	0.10	0.10	0.10	0.10	0.10	0.00
**Tryptophan**	0.00	0.00	0.10	0.00	0.10	0.00	0.10	0.00
**Choline—70%**	0.00	0.00	0.00	0.00	0.10	0.10	0.10	0.10
**Vitamins and trace mineral premix^b^**	0.20	0.20	0.20	0.20	0.20	0.20	0.20	0.20
**Other^c^**	0.60	0.60	0.60	0.50	0.50	0.50	0.50	0.50
**Calculated nutrients**								
**NE, Mcal/kg^d^**	2.39	2.21	2.39	2.09	2.42	1.96	2.44	1.82
**Crude protein, %**	19.54	18.18	16.90	15.12	14.89	12.50	13.38	11.77
**Total Lys, %**	1.41	1.29	1.18	1.06	1.06	0.82	0.94	0.71
**SID Lys, %^e^**	1.20	1.10	1.10	1.00	0.90	0.70	0.80	0.60
**SID Lys:Mcal NE**	4.95	4.95	4.20	4.20	3.55	3.55	3.15	3.15
**STTD phosphorus^f^**	0.40	0.40	0.40	0.40	0.40	0.40	0.40	0.40
**Calcium:Phosphorus**	1.20	1.20	1.20	1.20	1.20	1.20	1.20	1.20

Nutrients are reported on an as-fed basis. Dietary phase was changed approximately every 4 weeks starting at 9 weeks of age.

a Thr-Pro which provides Thr at 85% of product (CJ BIo, Seoul, South Korea).

b Vitamin and trace mineral premix provided 9900.99 IU Vitamin A, 17.65 IU Vitamin D, 77.01 IU Vitamin E, 3.74 mg Vitamin K, 0.33 mg Biotin, 1.76 mg Folic acid, 3.30 mg Pyroxidine, 44.00 mb Niacin, 27.50 mg Pantothenic Acid, 8.15 mg Riboflavin, 2.20 mg Thiamine, 0.037 mg Vitamin B12 per kg, 20.02 mg Copper, 0.50 mg Iodine, 100.11 mg Iron, 30.00 mg Manganese, 0.300 mg Selenium, 120.13 mg Zinc, 2200 FTU phytase, 19.80 mg Xylanase, 0.30 mg Ethoxyquin per kg of diet. Manganese, selenium, and zinc were included as 50% organic and 50% inorganic forms.

c Other Alimet (Novus, St. Louis, MO) was provided at 0.10%, Salcurb (Kemin, Des Moines, IA) was provided at 0.30%, Defusion Plus (Cargill Animal Nutrition, Minneapolis, MN) was provided at 20%.

d Net energy.

e Standard ileal digestible.

f Standardized total tract digestible.

Soybean hull inclusion was increased every 4 weeks as the gilts changed dietary phases in the grow-finish period.

### Sample collection and analysis

On 111 days of age, vulva widths were measured on five gilts from each pen for a total of 150 gilts measured per treatment within one cohort. In order to determine which gilts were assigned to this portion of data collection, gilt tag numbers were entered into a computer and the 5 gilts were randomly selected from the cohort population per pen. Using a laminated standard piece of paper, three square openings were cut on the edges of the paper. Each square was labeled and cut according to the size, S (small, <27 mm), M (medium, 27–34 mm), L (large, >34 mm) (Romoser et al. 2020). Using this technique, the measurement tool could be held by the vulva to determine the correct size category. Vulvas that measured <27 mm were given a score of 1, 27–34 mm were given a score of 2, and >34 mm were given a score of 3.

On day 150 post-wean, blood samples were collected from 40 gilts from each treatment of the gilts in the vulva scoring group via a sterile jugular venipuncture using a 10 mL serum vacutainer tube (Becton Dickinson, Franklin Lakes, NJ). The tag numbers from the gilts that were identified for vulva scoring were entered into a computerized randomization program for assignment to the blood sampling. The blood was separated via centrifugation (2000 × g for 10 min at 4°C) and the serum was collected and stored at −80°C for subsequent analysis of progesterone and 17β- estradiol.

On day 164 post-wean, blood samples were collected from the same pigs, the pigs were individually weighed using the digital platform scale at the gilt developer unit, and back fat and loin depth at the last rib from each pig was measured via ultrasound machine (EXAGO, IMV Technologies, Brooklyn Park, MN). Gilt growth rate was calculated from estimated birth weight to day 164.

### Enzyme-linked immunoassays for hormones

Concentrations of progesterone and 17β- estradiol were determined via enzyme-linked immunoassay (ELISA) on the principle of competitive binding. DRG Estradiol ELISA (Catalog #EIA2693, DRG International, Springfield, NJ) had a range of 10.6–2000 pg/ml and a sensitivity of 10.6 pg/ml. DRG Progesterone ELISA (Catalog #EIA1561, DRG International, Springfield, NJ) had a range of 0 to 40 ng/ml and a sensitivity of 0.045 ng/ml with an expected intra-assay variation of 6.42% and inter-assay variation of 6.63%. These analyses were performed using the manufacturer’s protocol.

### Dietary analysis

Soybean hull water holding capacity (WHC) was measured using the modified procedure of Giger-Reverdin (2000) as described by Ruckman et al. (2020). Feed samples were collected from each dietary phase for each cohort from the barn (feed core grabs from five feeders within each room) and stored at 0°C until further analysis. Samples from all cohort collections for an individual phase were combined and subsamples for further dietary analysis. Feed and soybean hull samples were sent to the University of Missouri Agricultural Experiment Station Laboratories (Columbia, MO) for total dietary fiber analysis and complete amino acid profile. Total dietary fiber and acid detergent fiber were analyzed using the standardized AOAC methods 985.29 (2006) and 973.18 A-D (2006). The amino acid profile and crude protein was determined using cation-exchange chromatography coupled with post-column ninhydrin derivatization and quantification (method 982.30 E; 988.15; AOAC, 2006; AOAC 990.03, 2006). Crude fiber and trypsin inhibitor (TIU) analysis were conducted using the AOAC 978.10, 2006 and AACC 22–40, 2006 methods. Starch was analyzed using the AACC 76-13 and modified with the Sigma (St. Louis, MO) starch assay kit (STA-20). Crude fat was analyzed using ether extraction (AOAC 920.39, 2006).

Feed samples were analyzed at Iowa State University for dry matter (DM), ash, crude protein, crude fat, and gross energy (GE). Diets were ground to 1 mm particle size with a Wiley Mill (Variable Speed Digital ED-5 Wiley Mill; Thomas Scientific, Swedesboro, NJ) and analyzed in duplicate. Dry matter and ash were calculated by the mass difference after oven drying at 100° C for 12 hours and at 600° C for 12 hours, respectively [method 930.15 (AOAC, 2007)]. Gross energy was measured using an isoperiodic bomb calorimeter (model 6200; Parr Instrument Company, Moline, IL). Benzoic Acid (6318 kcal/kg) was used as the standard for calibration and was determined to contain 6318 ± 18 kcal/kg. Crude protein was measured by the concentration of nitrogen [*N*, method 990.03 (AOAC, 2007); TruMac; LECO Corp., St. Joseph, MI]. An EDTA sample (9.56% *N*) was used as the standard for calibration and was determined to contain 9.55 ± 0.01% *N*. Crude protein was calculated as *N* × 6.25.

### Calculations and statistical analysis

Back fat and age were adjusted according to published equations (Hubbard 1981). Back fat was adjusted based upon weight of the female at the time of measurement to eliminate bias of weight on body composition. Age to the predetermined body weight was standardized to best assess growth rate to a select weight rather than time. Mating to farrowing interval was calculated by the difference between the age at farrowing and the age at first mating to determine the amount of gilts that required a second service. Total born, number of pigs weaned per sow, and pre-weaning mortality were recorded on farm.

The UNIVARIATE procedure in SAS 9.4 (SAS Institute, Cary, NC) was used to verify normality. A power test was conducted to determine the sample size for total born, body weight, vulva width and hormone assays. The room was the experimental unit and the farm was used as a random effect. The gilt was considered the unit of analysis. Confounders such as time of year during the growth period and also breeding period were tested. Statistical outliers were identified as data points greater than three standard deviations of the studentized residuals. The data associated with the outlier was excluded from the analysis. Back fat, loin depth, body weight, days of age, and sow performance were analyzed using proc GLIMMIX procedure. Progesterone and 17β- estradiol concentrations were analyzed using proc GLIMMIX using repeated measures. Since serum hormones were tested multiple days, the interaction of day and treatment were evaluated for progesterone and 17β- estradiol concentrations. Differences were considered statistically significant if *P* < 0.05.

## Results

### Diet analysis

The analysis of the soybean hull samples showed an ADF value of 51% and an amylase- treated neutral detergent fiber (NDF) value of 59.9% (Table 2). The WHC of soybean hulls ranged from 7% to 8%. Micron size of the soybean hulls used in the current study averaged around 690 ± 73.15 μm and included an average of 5.31 trypsin inhibitor units (TIU/mg). Dry matter was similar across dietary treatment and phase, but ash was higher for diets containing soybean hulls (Table 3). As expected, NDF was higher across the SG diets compared to the CON diets (Table 4). Insoluble and soluble fiber were also increased as the inclusion of soybean hulls increased. Neutral detergent fiber, insoluble fiber, and soluble fiber were lower in GDU 2 dietary phase than anticipated with the inclusion of soybean hulls.

**Table 2:** Ingredient composition of soybean hulls from ADM (Des Moines, IA) on a dry matter basis.

Item^a^	Soybean hull
**Crude protein, %**	11.09
**ADF^b^,%**	51.45
**aNDF^c^,%**	59.9
**Crude Fat,%**	1.35
**Starch, %**	0.31
**TIU^d^/mg**	5.31
**Particle size, μm**	690

a Acid detergent fiber and neutral detergent fiber were analyzed using the standardized AOAC method 973.18 A-D (2006) and JAOAC 56, p. 1352–1356, 1973. Crude fat and trypsin inhibitor (TIU) analysis were conducted using the AOAC 920.39, 2006 and AACC 22–40, 2006 methods. Starch was analyzed using the AACC 76-13 and modified with the Sigma (St. Louis, MO) starch assay kit (STA-20).

b Acid detergent fiber.

c Amylase-treated neutral detergent fiber.

d Trypsin inhibitor unit.

**Table 3:** Analyzed dietary composition for gilts fed low levels of soybean hulls (CON (0% added soybean hulls) or high levels of soybean hulls (SG (up to 50% added soybean hulls)) in the gilt developer unit (GDU).

		Dietary phase							
		GDU1		GDU2		GDU3		GDU4	
Item, %		CON	SG	CON	SG	CON	SG	CON	SG
**Proximate analysis^a^**									
	Dry matter	85.92	86.30	86.21	86.57	87.24	86.73	89.36	90.38
	Moisture	11.06	11.78	11.45	11.86	11.71	9.15	11.71	8.62
	Ash	5.49	4.12	4.98	4.47	4.34	5.00	4.24	5.07
	Crude Fat	2.52	4.09	3.78	3.84	3.77	3.34	3.76	3.06
	Crude protein	19.77	12.85	16.84	14.28	14.90	16.43	12.76	12.74
	Gross energy, Mcal/kg	3.86	3.87	3.86	3.87	3.86	3.89	3.93	3.98
**Total amino acids^b^**									
	Threonine	0.85	0.61	0.73	0.65	0.63	0.52	0.58	0.47
	Glutamic acid	3.43	2.35	2.82	2.68	2.50	1.93	2.22	1.75
	Proline	1.23	1.03	1.13	1.11	1.07	0.90	0.98	0.85
	Glycine	0.89	0.54	0.65	0.61	0.58	0.72	0.52	0.75
	Alanine	1.03	0.86	0.95	0.93	0.90	0.75	0.83	0.71
	Cysteine	0.35	0.27	0.29	0.28	0.27	0.26	0.26	0.25
	Valine	1.00	0.69	0.82	0.78	0.74	0.65	0.66	0.61
	Methionine	0.29	0.24	0.24	0.26	0.23	0.20	0.22	0.17
	Isoleucine	0.86	0.53	0.66	0.61	0.57	0.49	0.50	0.47
	Leucine	1.73	1.39	1.55	1.51	1.46	1.18	1.32	1.11
	Tyrosine	0.65	0.43	052	0.48	0.44	0.44	0.42	0.45
	Phenylalanine	0.97	0.65	0.79	0.74	0.70	0.58	0.62	0.54
	Lysine	1.27	0.88	1.04	0.94	0.92	0.86	0.83	0.73
	Histidine	0.51	0.35	0.42	0.40	0.37	0.34	0.34	0.32
	Arginine	1.18	0.70	0.91	0.83	0.75	0.62	0.68	0.58
	Tryptophan	0.16	0.13	0.17	0.14	0.13	0.09	0.12	0.06

Dietary phase was changed approximately every 4 weeks starting at 9 weeks of age.

a Proximate analysis of the diets was conducted at Iowa State University. Dry matter and ash were calculated by the mass difference after oven drying at 100°C for 12 hours and at 600°C for 12 hours, respectively [method 930.15 (AOAC, 2007)]. Gross energy was measured using an isoperiodic bomb calorimeter (model 6200; Parr Instrument Company, Moline, IL). Benzoic Acid (6318 kcal/kg) was used as the standard for calibration and was determined to contain 6318 ± 18 kcal/kg. Crude protein was measured by the concentration of nitrogen [*N*, method 990.03 (AOAC, 2007); TruMac; LECO Corp., St. Joseph, MI]. An EDTA sample (9.56% *N*) was used as the standard for calibration and was determined to contain 9.55 ± 0.01% N. Crude protein was calculated as *N* × 6.25. Crude fat was analyzed using the AOAC 920.39 (2006) method.

b The amino acid profile was determined using cation-exchange chromatography coupled with post-column ninhydrin derivatization and quantification (method 982.30 E; 988.15; AOAC, 2006; AOAC 990.03, 2006).

**Table 4:** Fiber analysis^a^ for each dietary treatment for gilts fed low levels of soybean hulls (CON (0% added soybean hulls) or high levels of soybean hulls (SG (up to 50% added soybean hulls)) in the gilt developer unit (GDU) by dietary phase.

	Dietary phase							
Fiber analysis Item, %	GDU1 CON	SG	GDU2 CON	SG	GDU3 CON	SG	GDU4 CON	SG
**NDF^x^**	12.24	18.57	10.56	14.88	11.80	31.32	11.58	38.86
**ADF^y^**	4.71	12.36	5.40	6.43	5.39	22.99	5.53	30.51
**Hemicellulose**	7.53	6.22	5.16	8.46	6.41	8.33	6.05	8.35
**Cellulose**	4.26	11.66	4.87	5.60	4.76	21.68	4.79	28.69
**Lignin**	0.40	0.69	0.49	0.90	0.63	1.44	0.84	2.30
**Starch**	28.25	26.46	26.20	26.61	34.34	32.42	31.81	20.34
**TDF** ^z^	17.86	26.64	16.77	20.62	18.15	34.22	17.13	44.52
**Soluble fiber**	1.11	2.01	0.92	1.81	0.98	1.86	0.83	1.59
**Insoluble fiber**	16.75	24.63	15.85	18.81	17.17	32.36	16.30	42.93

Dietary phase was changed approximately every 4 weeks starting at 9 weeks of age.

a NDF, ADF, hemicellulose, cellulose, lignin, starch, TDF, soluble fiber and insoluble fiber were analyzed at the University of Missouri. Methodologies were as follows: NDF (JAOAC 56, p. 1352–1356, 1973), ADF (AOAC 973.18, 2006), Hemicellulose (NDF-ADF), Cellulose (AOAC 973.18, 2006), Lignin (AOAC 973.18, 2006), Starch (AACC 76-13 with modification using Sigma (St. Louis, MO) starch assay kit (STA-20)), TDF/soluble fiber/insoluble fiber (AOAC 991.43).

x Neutral detergent fiber.

y Acid detergent fiber.

z Total dietary fiber.

### Growth performance

Gilts fed the CON diet had a higher average daily gain (ADG) compared to the SG group (*P* < 0.01; Table 5). Body weight (BW) at day 164 was higher for the CON treatment compared to the SG treatment group (*P* < 0.01). Adjusted days to reach 94 kg differed by nine days (*P* < 0.01) with the CON group reaching their target weight faster (155 days) compared to the SG group (164 days). Back fat was lower for gilts in the SG group than those in the CON group (*P* < 0.01). Loin depth was not different between dietary treatments (*P* = 0.57).

**Table 5:** Effect of dietary treatment on growth performance for gilts fed low levels of soybean hulls (CON (0% added soybean hulls) or high levels of soybean hulls (SG (up to 50% added soybean hulls)) in the gilt developer unit (GDU).

	Dietary treatment			
Items	CON	SG	SEM	*P-*value
**Number of gilts, *n***	150	150		
**Average daily gain, kg**	0.64	0.59	0.008	<0.01
**Body weight, kg**	102.2	94.8	2.26	<0.01
**Adjusted back fat, mm**	15.1	12.5	0.28	<0.01
**Loin depth, mm**	61.5	61.0	0.55	0.57
**Adjusted days to 94 kg**	155.4	164.3	1.39	<0.01
**Vulva width, mm**	2.94	1.58	0.054	<0.01

Vulva width samples were collected at 111 days post-wean and the body composition and growth data were collected at 164 days post-wean for one cohort within the study.

### Detection of reproductive development

Vulva widths differed between dietary treatment with a higher number of the CON gilts measuring >34 mm compared to a higher number of SG gilts measuring 27 to 34 mm (*P* < 0.01; Table 5). A serum 17β- estradiol concentration treatment by day interaction was observed (*P* < 0.01; Table 6), where 17β- estradiol concentrations on day 150 and 164 were higher in the CON group compared to the SG group.

**Table 6:** Effect of dietary treatment on serum hormone concentrations for gilts fed low levels of soybean hulls (CON (0% added soybean hulls) or high levels of soybean hulls (SG (up to 50% added soybean hulls)) in the gilt developer unit (GDU).

		Dietary treatment				*P*-value		
Items	Day	SEM	CON	SG	SEM	Treatment	Day	Trt*Day
**E2^a^**			30.77	18.44	0.903	<0.01	<0.01	<0.01
** Day 150**	27.70	0.860	37.28	18.13	1.216			
** Day 164**	21.50	0.894	24.25	18.74	1.281			
**P4^b^**			0.18	0.28	0.031	0.022	<0.01	<0.01
** Day 150**	0.11	0.030	0.14	0.07	0.043			
** Day 164**	0.35	0.030	0.21	0.49	0.042			

Forty gilts from each treatment were sampled from the same cohort that was evaluated for vulva size. Day listed in the table identifies the day of age for the gilt.

a E2:17β- estradiol.

b P4: Progesterone.

Additionally, a treatment and day interaction were observed for serum progesterone concentration (*P* < 0.01). Progesterone did not differ on day 150 between dietary treatment groups, but on day 164, the SG group had higher serum progesterone compared to the CON group at 0.49 ng/ml and 0.21 ng/ml, respectively.

### Sow performance

The first parity sow performance is presented in Table 7. The number of gilts that were sent to the sow farms were similar across treatments. Additionally, the number of gilts that the sow farms selected for breeding was similar across treatment and is reported as gilt retention. The number of gilts bred were also similar across dietary treatments. Days to first breed were 4 days longer for the SG group compared to the CON group but was not statistically different (*P* = 0.44). Farrowing rate was not significantly different with both the CON and SG. There was no difference between the CON and SG groups on total born (*P* = 0.43). There were no differences in total pigs born alive between the CON and SG group (*P* = 0.35). Total pigs weaned were similar between treatment groups (13.0 and 12.9, respectively; *P* = 0.81).

**Table 7:** Effect of dietary treatment on sow performance for gilts fed low levels of soybean hulls (CON (0% added soybean hulls) or high levels of soybean hulls (SG (up to 50% added soybean hulls)) in the gilt developer unit (GDU).

	Dietary treatment			
Item	CON	SG	SEM	*P*-value
**Gilts^a^, *n***	2266	2080		
**Gilt retention^b^, *n***	1836	1836		
**Bred gilts^c^, *n***	1604	1624		
**Days to first breed^d^, d**	227.7	231.8	4.09	0.44
**Farrowing rate^d^, %**	90.0	91.0	6.0	0.93
**Total born^d^, *n***	15.0	14.8	0.21	0.35
**Born alive^d^, *n***	13.9	13.7	0.20	0.43
**Total weaned^d^, *n***	13.0	12.9	0.24	0.81

a The number of gilts that completed the feeding trial from the grower to entry to the sow farm.

b The number of gilts from the study that were selected at the sow farm to be bred.

c The number of gilts from the study that were bred.

d These observations are from the number of bred gilts identified in this table.

## Discussion

Improving sow longevity in a herd is a key area for producers to improve lifetime productivity and parity age of the herd. By improving both of these parameters, the economic return per purchased female will also be increased resulting in improved sustainability for the swine industry. High growth rates during gilt development can lead to osteochondrosis which will contribute to leg weakness and lameness resulting in culling from the sow herd (Uhlhorn et al. 1995; de Koning et al. 2012). The objective of this study was to determine if the feeding of increasing levels of soybean hulls would slow the growth rate of a developing gilt without impairing first parity total born or conception rates.

This study was conducted at a singular gilt development facility that housed multiple rooms of gilts. Each room received a new group of gilts once a month. At the end of the grow-finish period, gilts were typically selected to be sent to sow facility based on fitting the guidelines for proper structure, teat count, and vulva parameters identified by the owner of the facility. Each month, the room of gilts were divided and sent to multiple sow farms within the ownership. This was repeated each month so that each treatment was represented at each sow facility in a similar timeframe. The delivery of both treatments to each sow farm within a similar timeframe allowed for potential health issues at sow farms, management differences across the sow farm, and seasonality to be equal across dietary treatments and reduce the sow farm management as a potential confounder of the data. In this study, the investigators then tracked the gilts to each sow farm to further evaluate downstream reproductive performance such as farrowing rate and total born.

During the course of the study, sow farms that would receive sexually mature gilts from this gilt developer facility experienced disease outbreaks, resulting in a portion of the gilts leaving the gilt developer facility prior to completing the dietary phases. This was done to allow for sow herd depopulation and closures to manage the disease outbreaks. These removals were in addition to grow-finish mortality, culls and non-selects being sent to harvest. Depending on when farms broke with disease, one cohort lost approximately 80% of the trial animals due to early shipments to the sow farms and two other cohorts lost approximately 50% of the trial animals. The cohort with more than 80% of animals removed was not included in the analysis. Animals from each room to be sent to the sow farm ahead of selection for entry into the reproductive herd around the time of puberty were identified by the farm staff with whole pens being sent to the sow farm and then the animals remaining in the room being reallocated to increase space allowances for the remaining females through the grow-finisher period. Approximately 100 gilts from each of the remaining rooms were sent to other isolation facilities prior to the end of the grower period and were not counted in the study.

The average wean to finish mortality of the gilt development during the trial period was approximately 4% across the cohorts. Some full-value gilts from the cohort were sent to market based upon sow farm needs while other gilts were identified as not matching the criteria for the reproductive female and were sent to market. The percent of animals identified as reproductive females from the population at the end of the grow-finish period was approximately 82%. Due to gilts being removed from the gilt developer unit before puberty to the sow farms, the wean to finish death loss, and average percent of animals meeting reproductive criteria for the sow farm, the total number of eligible females for the trial analysis was 4346 gilts.

In the current study, the formulated standard ileal digestibility (SID) Lys:NE was identical within dietary treatments across production phases. By formulating diets on a SID Lys:NE basis, the energy was allowed to fluctuate with fiber inclusion to naturally dilute energy without the extra inclusion of oil or other energy-dense ingredients to balance energy concentrations. The SG diets were formulated to have a lower lysine density than the control diets; however, the diets were balanced on the SID Lys:NE to optimize lean deposition based on energy intake. Allowing energy to decline as soybean hull inclusion would be a common practice in swine diets when energy sources (eg corn oil, soybean oil, choice white grease) are not economical to include. Since this was a commercial facility and the price of fat sources was not economical, fat was not added into the diet to hold energy constant. In addition, the use of oil or fat could have altered the growth responses and puberty onset in gilts due to the potential different in polyunsaturated fat:saturated fat ratios in the diets (Petrone et al. 2019; Becker and Greiner 2023). The SID Lys level of the SG diet was calculated to meet the requirements of the animal at that time. All other nutrients were also met in the SG diet. The CON animals were thereby fed a diet that was higher in Lys than their requirements. This type of diet formulation was done to reduce the potential impacts of reducing Lys and other nutrients and confounding the results. However, because energy was reduced when the fiber source was added, the results within this paper cannot be confirmed to only be associated to added fiber.

The inclusion level of soybean hulls up to 50% was required in order to continue to slow the growth of the gilt. Data from multiple studies evaluating high fiber diets are conflicting in terms of the impact on reducing feed intake particularly when fiber is fed over long periods of time (Powley et al. 1981; Varel et al. 1984; Pond et al. 1988; Déru et al. 2020; Helm et al. 2021). Initial pilot tests of the diets (unpublished data) at lower levels of soybean hull inclusion did not result in reduced growth rates. The authors continued to increase soybean hull inclusion until a reduction in growth was noted in the facility and used those as the dietary treatments for this study.

The analyzed dietary values for amino acids did not show identical lysine inclusion values within a dietary phase compared to the reported calculated values. The variation of amino acids for laboratory analysis was within published values associated with this laboratory analysis (Cromwell et al. 2000). The feeding of soybean hulls, which is an insoluble fiber source, resulted in a reduced growth rate primarily through the reduction in daily energy intake. Previous studies demonstrated similar findings in that BW decreased as soybean hull inclusion increased up to 22.5% of the diet (Goehring et al. 2019; Miller et al. 2025). Goehring et al. (2019) evaluated pig growth rates when comparing soybean hulls or dried distillers’ grains and found that body weight decreased in three different experiments with inclusion of up to 20% soybean hulls with or without dried distiller grains in nursery pigs demonstrating that soybean hulls can have a significant impact on growth rates. The rate of BW differences was variable across all previously reported studies, as well as, the current study. The magnitude of the difference in the response BW among these experiments could be due to the varying inclusion level of the soybean hulls in the diet, the age of pigs, and the duration of the feeding period.

A secondary objective of this study was to evaluate if feeding higher levels of soybean hulls would alter body composition of the female at the time of puberty leading to changes in reproductive hormones. A single cohort of animals was used to assess the second objective. It is possible that results noted in this cohort may not be representative of the entire population. A single cohort was selected to reduce variation of season as the trial was conducted for one year. The cohort was a group of animals that were not subjected to any periods of heat stress during development and no signs of illness.

The assessment of gilt body condition involves evaluating body mass or composition, primarily fat, protein, and skeletal structure (Charette et al. 1996). Various methods, including visual assessment and palpation, can be inaccurate and subjective depending on the caretaker’s scoring skills (Charette et al. 1996). Correlations between back fat and weight underscore the importance of accurate body condition assessment (Maes et al. 2004). Ultrasound technology offers a more precise method for measuring back fat thickness and loin depth to aid producers in assessing animal body condition (Maes et al. 2004). Vulva width at 15 weeks of age is a valuable tool for assessing reproductive potential with increased inclusion of gilts into the breeding herd and a greater number of piglets produced through parity 2 when selecting gilts of larger vulva width (Romoser et al. 2020).

In the current study, the feeding of soybean hulls decreased back fat depth by 2.6 mm, but did not alter loin depth around the time of puberty. Gregory et al. (2023) documented similar findings with a decrease in back fat in gilts fed either a restricted dietary program or a high fiber diet compared to the control at the time of breeding. The decrease in back fat also aligns with a similar response when gilts were fed a restricted diet at different levels of the control diet compared to the ad libitum fed gilts indicating that the reduction in daily caloric intake reduces back fat deposition (Klindt et al. 2001).

The findings of this study on the impact of increasing soybean hulls on loin depth and carcass composition is contradictory to other published work. The feeding of increasing amounts of soybean hulls at 0, 7.5, 15, and 22.5% in grower pigs, resulted in carcass weight, carcass back fat, and loin depth decreasing linearly (Miller et al. 2025). Goehring et al. (2012) conducted a study with grower pigs over the course of 118 days with the inclusion of either 7.5 or 15% soybean hulls ground or unground, observed that increased inclusion of soybean hulls lowered carcass yield, hot carcass weight, and back fat depth, which resulted in a linear increase in percent lean and fat free lean index as the inclusion of soybean hull increased. Pigs fed a 30% soybean hulls or wheat middling’s diet in the finishing phase had a lower hot carcass weight and dressing percentage compared to the control group (Stewart et al. 2013). The reduction in hot carcass weight and dressing percentage is likely due to the increase in visceral weight associated with increased gut fill and capacity that results in reduced energy directed towards fat and lean deposition (Agyekum and Nyachoti 2017). The lack of response of the protein deposition relative to soybean hull inclusion in this study is unclear and warrants further investigation. Back fat levels have a direct influence on puberty across multiple species. Leptin is produced in fat cells and is believed to be an important signal to initiate puberty (Kaplowitz 2008). The existing body of work demonstrates that the feeding of high levels of soybean hulls will reduce back fat levels in swine, but little work has been conducted to evaluate if the reduced body fat altered reproductive hormones in the gilt. Vulva scores were higher in the CON group compared to the SG group indicating that the CON gilts were more sexually mature at day 111 compared to the SG group. Beyond the measurement of the vulva, the measurement of circulating hormones at two points were conducted. The timepoints were selected based upon when a boar would be introduced to the gilts. The first timepoint (day 150 post-wean) is prior to boar exposure and the second timepoint was one week after boar exposure was initiated.

17β- estradiol and progesterone are two hormones that can be utilized to identify puberty onset in gilts, as higher 17β- estradiol indicates the onset of puberty in the animal. Progesterone concentrations are lower (<1 ng/ml) during estrus but begin to increase after day 2, until reaching peak values between day 8 and 12 of the cycle (Henricks et al. 1972). 17β- estradiol circulates in the blood until it reaches the liver, where it is metabolized and transported in bile for secretion or into peripheral circulation (Ruoff and Dziuk 1994; Anderson, 2009). When Zhuo et al. (2017) fed gilts either soluble fiber or minimal fiber, progesterone concentrations measured at day 205 were not different, but soluble fiber resulted in higher concentration of 17β- estradiol in the control group on day 205. Additionally, 17β- estradiol and progesterone levels are higher during the estrus cycle in gilts that had already expressed two estrous cycles (Henrick et al. 1972). Additionally, 17β- estradiol levels fluctuate during the estrus cycle in which 17β- estradiol increased one to two days prior to estrus (>20 pg/ml) (Henricks et al. 1972). In this study, there was an elevated level of 17β- estradiol for both sample days indicating that the CON gilts were cycling at a higher level than the SG group and that soybean hulls did not increase 17β- estradiol. Additionally, a day response for progesterone was noted at day 164 indicating that the gilts had expressed their first initial estrus. Furthermore, there was a diet by day interaction indicating that the CON gilts were expressing higher levels of progesterone at day 150 compared to the SG group and then the SG group had a higher level of progesterone than the CON at day 164.

With the detected reduction in back fat and altered indicators associated with reproductive maturation, the authors evaluated first parity retention to first breeding, first parity farrowing rate and total born. The farrowing rate and total born per sow was unchanged based on soybean hull inclusion indicating that feeding soybean hulls and reducing growth rate did not influence the gilt’s total born or farrowing rate. Similar to the results in this study, Zhuo et al. (2017) found no difference between gilts fed a diet with insoluble fiber (wheat bran) and the control group on total born alive. Additionally, Gregory et al. (2023) reported no difference between dietary treatment on total born alive when feeding a high fiber diet, restricted diet, or a control diet. Feed intake management does not appear to alter performance in that gilts fed either ad libitum, 90% of ad libitum or 74% of ad libitum from 13 to 25 weeks of age had no differences in days to first estrus or litter performance parameters across dietary treatments (Klindt et al. 2001). The lack of response into the first parity would indicate that a nutrition program that meets the gilt’s needs during gestation is sufficient to support embryonic development. Similar to other research studies, this study noted little influence on feed restriction during gilt development and subsequent reproductive performance (Stalder et al. 2000; Klindt et al. 2001). The addition of soybean hulls did not improve sow retention in the herd through first parity. Further tracking of these females will need to be done to determine long term longevity in the herd.

## Conclusion

Under the conditions of this study, the inclusion of dietary insoluble fiber in the form of soybean hulls reduced the energy concentration in the diet and resulted in reduced growth rate and fat deposition in prepubertal gilts, demonstrating its potential as a nutritional strategy for managing body weight and body composition during development. Feeding increasing levels of soybean hulls also resulted in a modest delay in the onset of puberty, which was associated with changes in vulva development and reproductive hormone profiles. Despite these effects on growth and pubertal development, reproductive performance through first parity was maintained, indicating that the use of soybean hulls in gilt developer diets can modify growth and maturation patterns without compromising subsequent reproductive success.

## List of abbreviations

ADF: acid detergent fiber
aNDF: amylase-treated neutral detergent fiber
ADG: average daily gain
BW: body weight
CON: control
DM: dry matter
ELISA: enzyme linked immunoassay
FASS: Federation of Animal Science Societies
FTU: phytase units
GDU: gilt developer unit
GE: gross energy
IACUC: Institutional Animal Care and Use Committee
Lys:NE: lysine to net energy
NDF: neutral detergent fiber
NE: net energy
SG: slow growth
SID: standard ileal digestible
STTD: standardized total tract digestible
TDF: total dietary fiber
TIU: trypsin inhibitor units
